# 1/2 order subharmonic waves of two cavitation bubbles

**DOI:** 10.1016/j.ultsonch.2024.107022

**Published:** 2024-08-19

**Authors:** Feng Tao, Guo-ying Zhao, Wei-zhong Chen, Duo Tao

**Affiliations:** a School of Electrical and Information Engineering, Anhui University of Technology, Ma’anshan 243002, China; b School of Computer Science and Technology, Anhui University of Technology, Ma’anshan 243002, China; c Key Laboratory of Modern Acoustics, Ministry of Education, and Institution of Acoustics, Nanjing University, Nanjing 210093, China

**Keywords:** Subharmonic, Mutual interaction, Cavitation bubble, Bubble energy

## Abstract

In the work, the 1/2 order subharmonic wave of two coupling cavitation bubbles is investigated numerically via Fourier spectrum analysis. By analyzing the dynamics of bubble, we find that the mutual interaction between bubbles can affect the appearance of 1/2 order subharmonic. The results of parameter dependence show that the intensity of 1/2 order subharmonic would be promoted or inhibited with the increase of mutual interaction. The higher the driving amplitude or the smaller the distance between bubbles, the stronger the mutual interaction is, and also the greater the promotion or suppression of the 1/2 order subharmonic is. Moreover, while the 1/2 order subharmonic occurs, the energy of bubble would alternate between two different peaks, and the temperature inside bubble has a similar fluctuation while the bubble collapses. This qualitative analysis suggests that the bubble′s dynamics for multi-bubble case is complex. Understanding the generation of subharmonic of bubble′s dynamics is of great significance for helpful applying of cavitation bubble.

## 1 Introduction

Since the pioneering work of Lord Rayleigh [1], cavitation as a very typical hydrodynamic phenomenon has received a great amount of attention. Cavitation bubble has complex behavior, meanwhile, it is also very difficult to observe in the engineering field for its small size and short life cycle directly. However, as an interesting and significant issue, both experimentally and numerically, acoustic cavitation generated by ultrasonic field has been extensively studied and many articles have been published over the years.

The study of cavitation bubble mainly has two aspects: single bubble and multi-bubble. Obviously, in addition to the influence of the inherent properties of liquid, such as viscosity, for single bubble case, the dynamics of bubble is only affected by the driving sound field. In Refs. [2], [3], [4], [5], [6], [7], [8], the researchers have investigated the stable dynamics of single bubble in detail and found the peak of pulsation is constant throughout all driving sound cycles. Furthermore, in the investigation of sonoluminescence, the brightness period of bubble at the time of collapse is also consistent with the driving sound period. However, under the same condition of circumstances, for multi-bubble case, the dynamics of each bubble becomes much more complex, which is affected not only by the driving sound field, but also by the sound pressure radiated from the surrounding bubbles. Considering the complex interaction between many cavitation bubbles, the nonlinear oscillations of multi-bubble have been analyzed by many predecessors [9], [10], [11], [12], [13], [14], [15], [16], [17], [18], [19], [20], [21]. For instance, in Ref. [21], the mutual interaction between bubbles was carefully investigated and the cavitation noises emitted in different sonication conditions were recorded to study the dynamical behavior of bubbles. The corresponding results suggest that the oscillations of bubbles could be severely influenced by the dispersing state of bubbles, and that the nonlinear feature of the dynamics of cavitation bubbles, imposed by the mutual interaction between bubbles, would be gradually developed with the decrease of the dispersing height.

As we all know, despite the numerous studies that shed light on the multi-bubble dynamics, due to the complexity of the system, the bubble behavior is not yet fully understood, such as doubling period and chaos, and so on. Fortunately, up to now, some researchers have carried out beneficial exploration in this aspect. Like the logistic and the Lorenz systems, as one of the typical nonlinear systems, the cavitation bubble is in deed typically characterized by the complex behavior of bifurcation and chaos. Author [[22]] has investigated the chaos behavior of bubble oscillations by using many methods, such as Lyapunov exponent, Poincaré map and phase diagram, *etc. Similarly, in Behnia and co-workers*′ *researches, the periodic and chaotic of bubble have been investigated by controlling specific ranges of parameters* *[*23*]**, and the suppressing chaotic oscillations of a spherical cavitation bubble is probed through applying a periodic perturbation* *[*24*]**. Sojahrood et al. has used a comprehensive bifurcation analysis method to study the nonlinear radial oscillations of the bubble oscillator by sonicating with different resonance frequency* *[*25*]**.*

From regular motion to chaos, an interesting transition, period doubling which is concomitant with the generation of 1/2 order subharmonic (SH) has always played a key role in the dynamical system. For stable cavitation, 1/2 order SH as an indicator has been used in many aspects, such as monitoring treatments [26] and BBB opening [27], and so on. It is known that, for single bubble case, as the driving pressure increases, the nonlinear response could become chaotic and the bubble radius would grow beyond a limit that may lead to bubble destruction finally. However, the mechanism of period doubling still needs to be further investigated, especially in the case of multi-bubbles, due to the interaction between bubbles, this phenomenon would become more complex. Based on this consideration, this article would study the generation of 1/2 order SH in multi-bubble case. In fact, the larger the number of bubbles in liquid, the more difficult the study of bubble dynamics is. In the investigation, we would use the two-bubble model widely applied in many literature where it is recognized as the simplest representation for multi-bubble case, to study the relationship between the occurrence of 1/2 order SH and the interaction qualitatively without considering the translation of bubbles.

The article is organized as follows: In Section 2, we present the sketch of two coupling spherical bubbles and the corresponding equation which is used to investigate the dynamics of bubble. In Section 3, the numerical calculations for the two-bubble case are shown, which investigate the generation of 1/2 order SH via Fourier spectrum analysis. In order to help to understand the effect of mutual interaction between bubbles on the intensity of 1/2 order SH, the parametric dependencies of the 1/2 order SH would be investigated in Section 4 with different driving amplitude and different distance between bubbles. In Section 5, the temperature inside cavitation bubble and the energy of bubble are discussed while the 1/2 order SH occurs. In Section 6, there is a conclusion and discussion.

## 2 Theoretical model

**Fig. 1:**
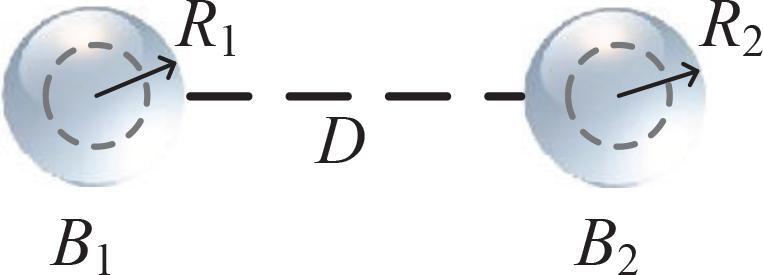
Sketch of two coupling spherical bubbles. $R_{i}$ is the instantaneous radius of bubble labeled by $B_{i}$ (i=1,2).

Fig. 1 gives a sketch of two cavitation bubbles 1 and 2 which are labeled by $B_{1}$ and $B_{2}$ respectively. $B_{2}$ can be seen as a neighbor of $B_{1}$, and vice versa. In this research, bubbles are subjected to driving ultrasound with the conventional single frequency source,

(1) $$P_{\mathrm{dr}}=p_{a}\sin2\pi \mathrm{ft},$$

with amplitude $p_{a}$ and frequency *f*.

The dynamics expression of *i*-th spherical bubble with the instantaneous radius $R_{i}$ in a liquid is usually described by Keller-Miksis equation [19], [15], [21], a modified Rayleigh-Plesset model,

(2) $$\begin{array}{cc} & \left(1-\frac{{\overset{˙}{R}}_{i}}{c}\right)R_{i}{\overset{¨}{R}}_{i}+\frac{3}{2}\left(1-\frac{{\overset{˙}{R}}_{i}}{3c}\right){\overset{˙}{R}}_{i}^{2}\\ & =\frac{1}{\rho }\left[\left(1+\frac{{\overset{˙}{R}}_{i}}{c}\right)P_{i}+\frac{R_{i}}{c}\frac{{\mathrm{dP}}_{i}}{\mathrm{dt}}+P_{\mathrm{rad}}^{\left(3-i\right)}\right],(i=1,2) \end{array}$$

where the over-dots mean the derivative respect with the time *t*. The parameters ρ and *c* express the density and sound speed of the liquid, respectively. And the pressure $P_{i}$ near the *i*-th bubble wall is given by,

(3) $$P_{i}=p_{i,b}-\frac{2\sigma }{R_{i}}-\frac{4\mu {\overset{˙}{R}}_{i}}{R_{i}}-P_{\mathrm{dr}}-P_{0},$$

(4) $$p_{i,b}=\left(P_{0}+\frac{2\sigma }{R_{0i}}\right){\left(\frac{R_{0i}^{3}-h_{i}^{3}}{R_{i}^{3}-h_{i}^{3}}\right)}^{\gamma },$$

with σ and μ being the surface tension and viscosity of the liquid. $P_{0}$ is the hydrostatic pressure. For the *i*-th bubble, the symbols $R_{0i}$ and $h_{i}$ represent the equilibrium radius and the hard-core radius of van der Waals gas ($h_{i}=R_{0i}$/8.86 for argon [28] which is used in the following investigation.), respectively. The polytropic exponent γ=5/3 describes the thermal conduction procedure of the adiabatic argon gas inside the bubble.

In Eq. (2), $P_{\mathrm{rad}}^{\left(3-i\right)}$ represents the radiative pressure [29] from the neighbor of *i*-th bubble. For the case of single bubble, this term should be ignored.

(5) $$P_{\mathrm{rad}}^{\left(3-i\right)}=-\frac{\rho }{D}\left(2R_{3-i}{\overset{˙}{R}}_{3-i}^{2}+R_{3-i}^{2}{\overset{¨}{R}}_{3-i}\right),$$

where $R_{3-i}$ is the neighbor′s radius of *i*-th bubble. The parameter *D* is the distance between two bubble centers.

In present investigation, all bubbles are assumed to retain its sphericity, and the vapor pressure and mass exchange are not taken into account. Because the pulsating of bubbles is investigated in a fixed position, the translational motion of bubbles which induced by the secondary Bjerknes force between them is not considered.

During the pulsation, the temperature inside the bubble is constantly changing. The temperature $T_{\mathrm{in}}^{\left(i\right)}$ inside *i*-th bubble can be calculated by [30]

(6) $$T_{\mathrm{in}}^{\left(i\right)}=\frac{4\pi p_{i,b}\left(R_{i}^{3}-h_{i}^{3}\right)}{3N_{t}k}$$

where $N_{t}$ is the number of molecules in the bubble, and *k* represents the Boltzmann constant, respectively.

In the paper, Eq. (2) would be calculated to investigate the dynamics of the two bubbles by using the fourth Runga–Kutta routine in the standard MATLAB adaptive solver ode45 which has a fifth order error estimation. During investigating, we assume that the pure water is host liquid, and the calculations are carried out applying the constant values for $c=1.50\times 10^{3}$ m/s and $\rho =1.00\times 10^{3}$ kg/m^3^, $\sigma =7.28\times 10^{-2}$ N/m, and $\mu =1.002\times 10^{-3}$ kg/m·s. Unless otherwise stated, the driving amplitude $p_{a}$ of sinusoidal sound is set to be 1.35 atm (1 atm ≈ 1.01 × $10^{5}$ Pa). To avoid transient oscillations and obtain a stable resolution in the frequency spectrum, for each simulation parameter, all analyses are performed within the last 100 cycles of a 1000 cycle acoustic pulse.

As a valuable method, the Fourier spectrum analysis is usually used to analyze the dynamics of nonlinear system, which can present the relation between the changes of dynamics of system and a wide range of control parameters effectively. In the work, we introduce this way to present the evolution of 1/2 order SH affected by mutual interaction between bubbles qualitatively.

## 3 The 1/2 order SH wave of two cavitation bubbles

The dynamic behavior of bubble is known to be affected by its coupled bubble, which is reflected in the interaction between bubbles. In this section, we would investigate the generation of 1/2 order SH, f/2, of two coupling cavitation bubbles. In the investigate, the spectrum structure only contains the 1/2 order SH and its SHs, beside that the fundamental frequency component.

**Fig. 2:**
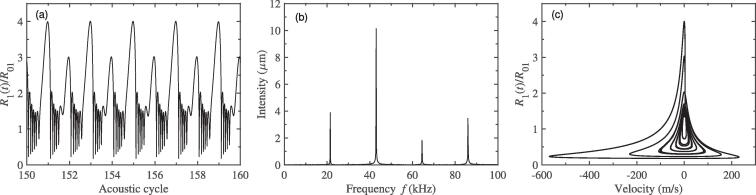
The change in bubble radius for the two-bubble case. (a) the radial pulsation of bubble 1; (b) the corresponding frequency spectra of (a); (c) the phase portrait diagram corresponding to (a). The equilibrium radii of two bubbles 1 and 2 are both 10μm, and the driving frequency is 43 kHz.

**Fig. 3:**
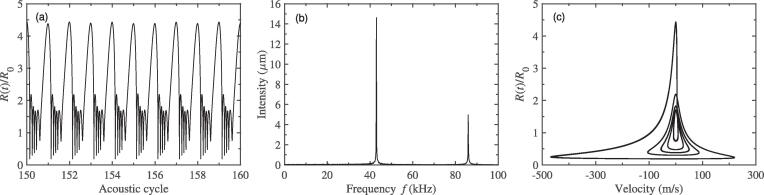
The change in bubble radius for the single bubble case. (a) the radial pulsation of bubble; (b) the corresponding frequency spectra of (a); (c) the phase portrait diagram corresponding to (a). The equilibrium radius of bubble is 10μm, and the driving frequency is 43 kHz.

Here, the distance between two bubble centers, *D*, is set to be 150μm, and the two bubbles would be exposed to a 43 kHz external driving pressure field. Fig. 2(a) plots the pulsation of bubble 1 with respect to the acoustic cycles while the ambient radii of bubble 1 and 2 are $R_{0i}=10\;\mu$m (i=1,2). It is easy to see that, as time goes on, two different peaks alternate on the pulsation curve. This phenomenon indicates that, besides the fundamental frequency *f*, additional frequency component is produced. In Fig. 2(b), the spectrum corresponding to the radius-time variation of Fig. 2(a) is given. The generation of half the driving frequency component, namely f/2 SH, has been observed. That is to say, the period doubling occurs during bubble pulsation. Due to the same equilibrium radius, the pulsation of bubble 2 is similar to that of bubble 1 and is no longer given here. It is well known that, when the bubble pulsates in the sound field, it will produce radiation pressure which can be regarded as a secondary sound source. While the distance of two bubbles is small, their radiation pressures can influence each other′s dynamics. Therefore, we believe that the appearance of SH is related to the radiated sound pressure between bubbles. In order to illustrate this view, here, the pulsation curve, for the single bubble case, is also given and the corresponding spectrum is calculated. From Fig. 3(a), we can see that the bubble can pulsate in a stable cavitation state in which the bubble expands during the rarefaction phase and collapses at the compression phase of ultrasound field, and the peak value of pulsation is the same in each driving cycle. It shows that, in the case of single bubble, there is no SH component would be created, which could be understood clearly from the corresponding frequency spectrum in Fig. 3(b) only containing fundamental frequency and its superharmonics. As one can imagine, for the two-bubble case, further increasing in value of distance between bubble centers, the pressure acting from one bubble on another would become small, the SH component would gradually become weaker and weaker, and finally disappear. Therefore, the mutual interaction between bubbles which is resulted by the radiation pressure is the cause of SH is quite reasonable. Here, to further examine the dynamics of period doubling in Fig. 2(a), Fig. 2(c) shows the phase portrait of the bubble 1 for the two-bubble case. It is obviously that the phase diagram consists of a closed trajectory that passes two different peaks of pulsation of adjacent period, which is different from that in Fig. 3(c) for the single bubble case.

**Fig. 4:**
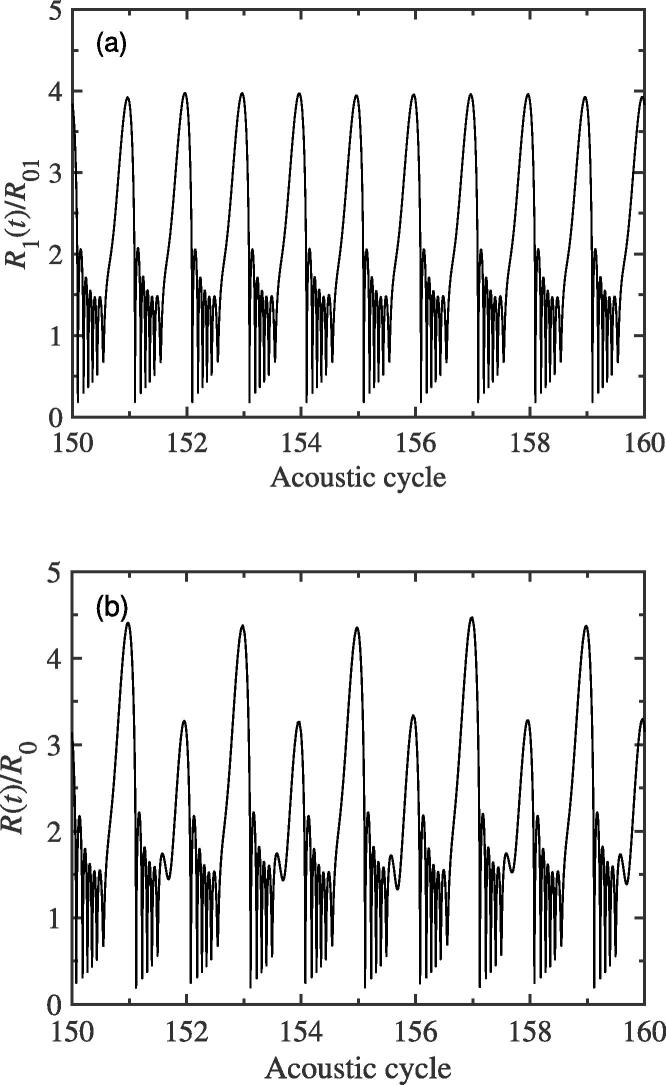
The pulsations of bubble (a) for the two-bubble case and (b) for the single bubble case. The equilibrium radius of bubble is 10μm, and the driving frequency is 39 kHz.

Of course, the interaction between bubbles does not always promote the generation of f/2 SH, and sometimes it plays a role in suppressing this SH. For example, when the driving frequency is set to be 39 kHz and other simulation parameters remain unchanged, the bubble′s pulsation for the two-bubble case has no f/2 SH component (see Fig. 4(a)). On the contrary, the pulsation of bubble for the single bubble case will contain the SH wave (see Fig. 4(b)). The corresponding spectrum diagrams of Fig. 4(a) and (b) are no longer given here. Therefore, we can conclude that the interaction between bubbles has two effects, that is, it sometimes promote the production of f/2 SH, and sometimes inhibit the appearance of f/2 SH.

**Fig. 5:**
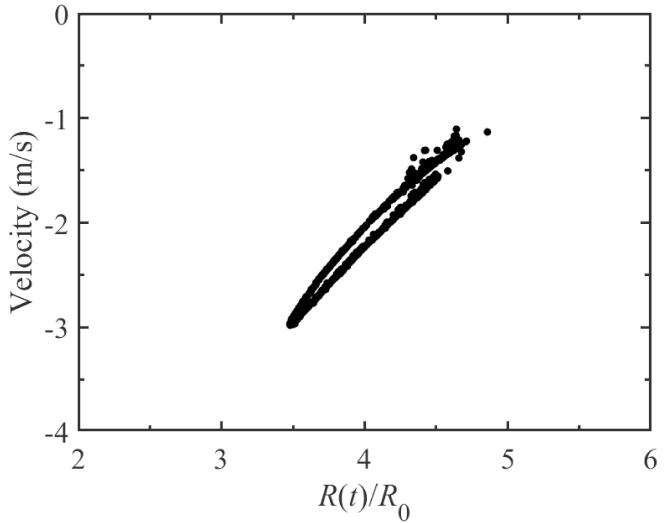
The Poincaré section for the single bubble at 35 kHz driving pressure field.

**Fig. 6:**
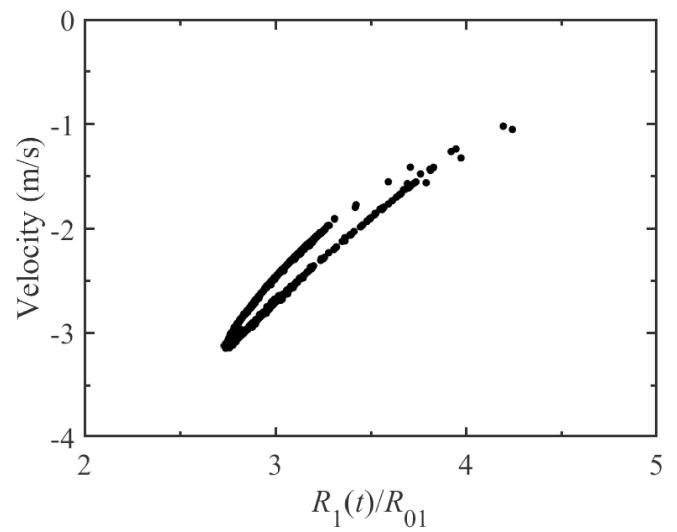
The Poincaré section of bubble 1 for the two-bubble case while the driving frequency is 46 kHz.

The figure of pulsation where f/2 SH occur is obviously characterized by period-doubling, which is likely to be signs of chaos. For instance, while the single bubble above is exposed to a 35 kHz driving pressure field, the radius-time curve behaves in a totally chaotic way and displays no periodical characteristic at all, which can be explained by that there are a lot of points scattered on the Poincaré section (see Fig. 5). Similarly, for the two-bubble case, the bubble pulsation will also appear chaotic. The Fig. 6 shows the Poincaré section for bubble 1 when the drive frequency is set to be 46 kHz. It is clearly that there is no period on the bubble’s pulsation.

## 4 The parametric dependencies of the 1/2 order SH

### 4.1 Effect of amplitude $p_{a}$ on f/2 SH

**Fig. 7:**
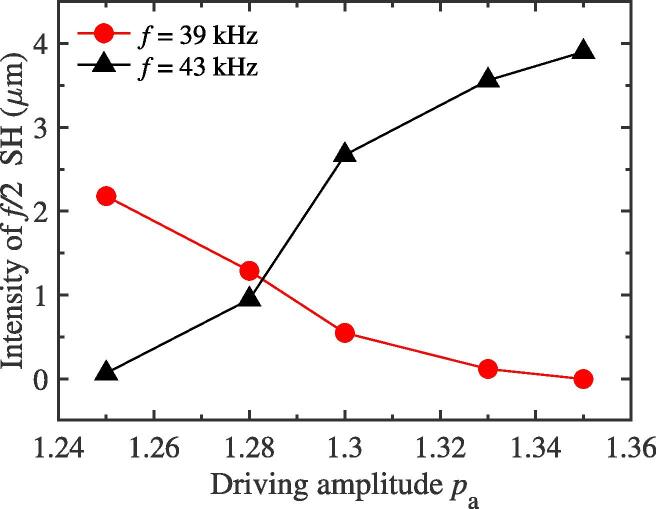
The plots of the f/2 SH intensity of one bubble′s pulsation for the two bubble case as the function of driving amplitude $p_{a}$ while the driving frequency *f* is set to be two different values.

**Fig. 8:**
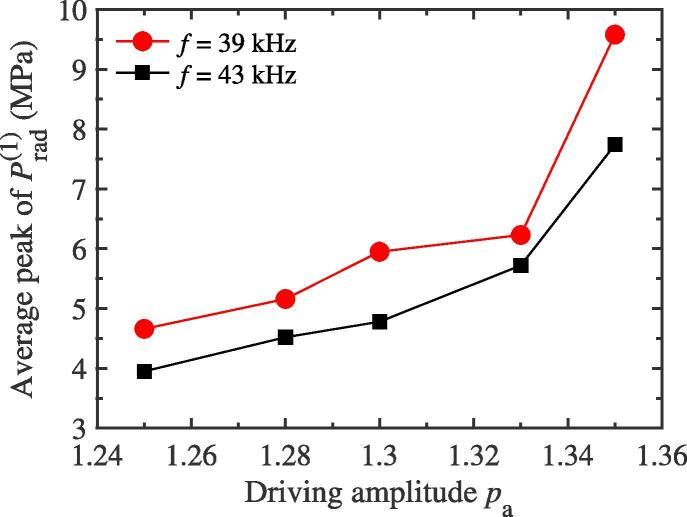
The relations between the average radiative pressure peak of bubble 1 and the driving amplitude $p_{a}$ with two different frequencies *f* for the two-bubble case. The radiative pressures are measured at 150 μm away from the center of the bubble 1.

**Fig. 9:**
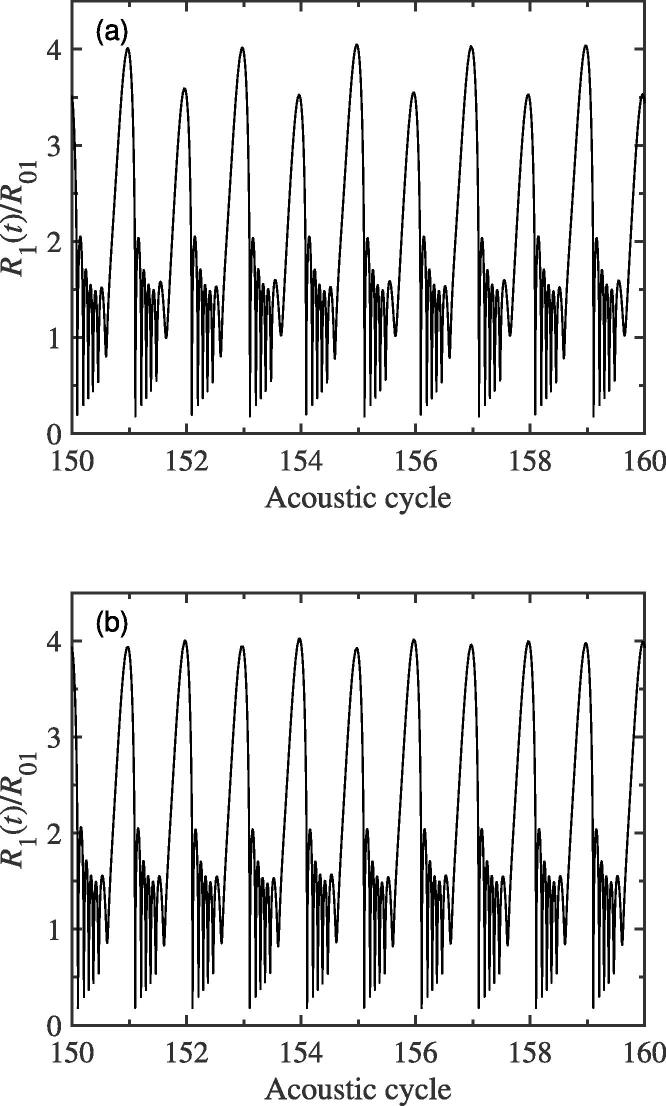
The pulsations of bubble 1 for the two-bubble case with driving frequencies (a) 42 kHz and (b) 41.4 kHz. The other parameters used to simulate are the same as those in Fig. .2(a).

Now, we will discuss the dependency of f/2 SH intensity on the driving amplitude $p_{a}$ for the two-bubble case. In this subsection, the equilibrium radius values of two bubbles used to simulate are the same as those of Fig. 2(a). The distance between two bubble centers remains constant, 150 μm, for different driving amplitudes. Fig. 7 gives the relations between the intensity of f/2 SH and the driving amplitude $p_{a}$ with two different driving frequencies 39 kHz and 43 kHz, respectively. It can be seen qualitatively that the f/2 SH is enhanced with the increase in the driving amplitude while the bubbles are exposed to a 43 kHz external driving pressure field. Conversely, it can be inferred that, at a certain small amplitude, this SH component will never be generated. However, when the driving frequency is adopted as 39 kHz, the intensity of 1/2 order SH would decrease with the increasing of driving amplitude (see red solid circle in Fig. 7), which is the opposite of the case at 43 kHz. This result shows that the mutual interaction between bubbles could also inhibit the generation of f/2 SH. As a matter of fact, while the driving amplitude, $p_{a}$, changes, the strength of mutual interaction between bubbles can be known from the variation of radiative pressure qualitatively. For bubble 1, Fig. 8 plots the changes of average peak of radiative pressure $P_{\mathrm{rad}}^{\left(1\right)}$ with the variation of $p_{a}$ at two driving frequencies 39 kHz and 43 kHz. It can be seen that the radiative pressure would become stronger and stronger with the increasing of $p_{a}$ gradually. Therefore, we believe that both the promotion and suppression degree of the intensity of 1/2 order SH for the two-bubble case are related to the increasing value of mutual interaction. As we all know, the pulsation of cavitation bubble could be changed by using different driving frequency. Fig. 9(a) and (b) show the radius-time variations of bubble 1 for two-bubble case with driving frequencies 42 kHz and 41.4 kHz respectively. It is clearly that, from Fig. 2, Fig. 4, Fig. 9, a small change in the driving frequency causes a large difference in cavitation bubble pulsation, which confirms the results in Fig. 7, that is, the two frequencies in Fig. 7 are not very different, but the intensity of f/2 SH are very different.

### 4.2 Effect of distance *D* on f/2 SH

**Fig. 10:**
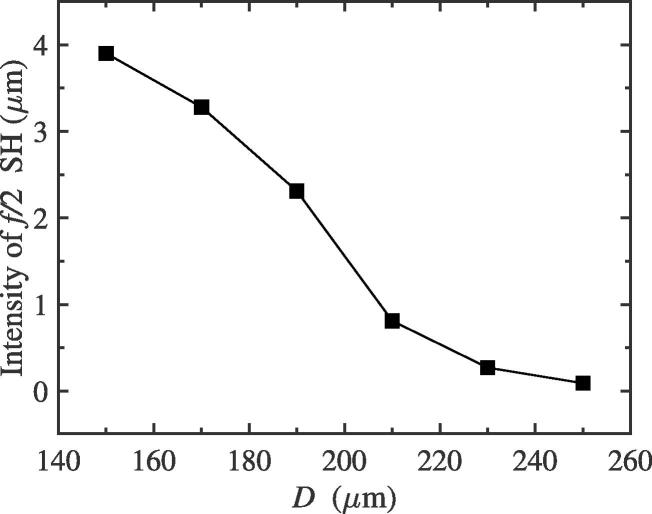
The intensity of f/2 SH with different distance between bubble 1 and 2 while the driving frequency is adopted as 43 kHz.

**Fig. 11:**
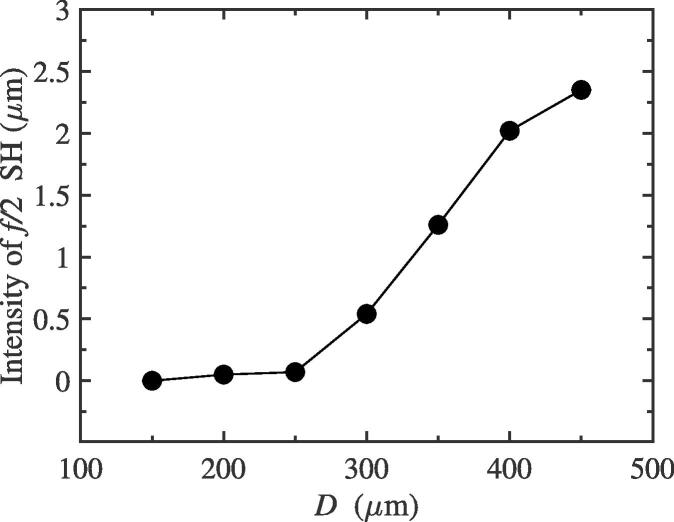
The relation between the intensity of f/2 SH and the distance between bubbles with the driving frequency 39 kHz for the two-bubble case.

Here, we would investigate the intensity change of f/2 SH by adjusting the distance between bubble centers *D* for the two-bubble case. The equilibrium radii of bubble 1 and 2 are all 10 μm. The driving amplitude, $p_{a}$, is adopted as that in Fig. 2(a). While the driving frequency, *f*, is set to be 43 kHz, from Fig. 10, we can see the intensity of f/2 SH gradually decreases as the distance between bubble centers increases. In fact, for one bubble, while the distance *D* become large, the radiative pressure exerted on it from another bubble would become weak, which can be understood from Eq. (5). Therefore, the mutual interaction between bubbles shows a decreasing trend. This result reflects that the 1/2 order SH intensity could be enhanced with the increase of mutual interaction between bubbles. However, when the two bubbles are exposed to a 39 kHz external driving pressure field, we find that the intensity of 1/2 order SH would increase as the distance between bubbles increases (see Fig. 11). It shows that the strong interaction, when the distance between bubbles is close, could inhibit the generation of f/2 SH, which is consistent with one of the conclusion of the previous subsection. The change of radiative pressure on the distance is no longer given here. The results in this subsection qualitatively verify again that the interaction between bubbles can enhance or inhibit the generation of f/2 SH.

## 5 Energy inside bubbles due to the generation of the 1/2 order SH

### 5.1 Temperature inside bubble

**Fig. 12:**
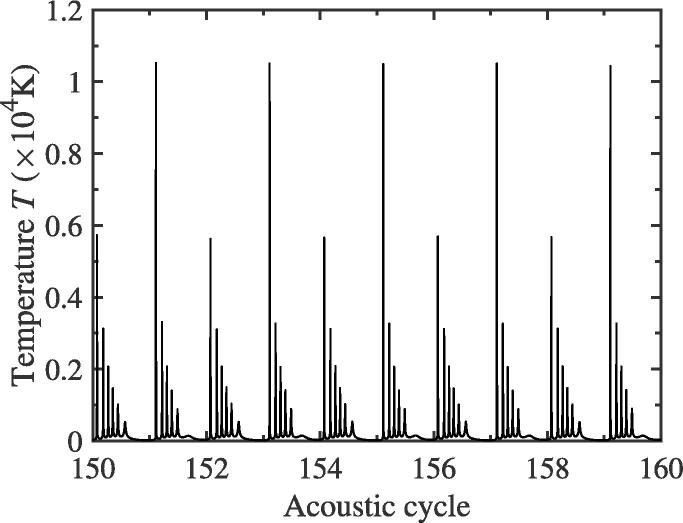
Collapsing temperature curve of bubble 1 for the two-bubble case. The equilibrium radii of two bubbles are both 10 μm. The driving amplitude and frequency, $p_{a}$ and *f*, are 1.35 atm and 43 kHz, respectively.

In this subsection, the change of temperature inside the bubble would be investigated qualitatively while the f/2 SH component generates. The calculating parameters are the same as those in Fig. 2(a). We assume that the temperature of host liquid, pure water, is $T_{0}=293$ K. Fig. 12 presents the change of instant temperature in the bubble for the two-bubble case. It is clearly that, as the acoustic cycle increases, the maximum of collapsing temperature inside bubble would alternate between two different peak values, which is similar to that of pulsation curve in Fig. 2(a). This can be easily understood that, because of the mutual interaction between bubble 1 and 2, the bubble′s pulsation in each acoustic cycle would be changed, which is labeled by the generating of f/2 SH. As we all know, while the pulsation is stronger, the temperature would be higher at the compression phase of ultrasound field. Therefore, we believe that, while the f/2 SH generates in the pulsation of bubble, the different temperature would lead to the difference of light bright in two consecutive acoustic cycles.

### 5.2 Energy of cavitation bubble

It is well known that, while a cavitation bubble pulsating in acoustic field, its volume undergoes periodic expansion and contraction. As a bubble expands from the equilibrium radius to the maximum one, the surrounding liquid will be displaced. For the two-bubble case, while the vapor pressure inside the bubble 1 is not taken into account, the work done by the liquid displaced by the expansion of bubble 1, $E_{b1}$, could be calculated by using Eq. (7) [31], which is also usually termed the energy of cavitation bubble 1.

(7) $$E_{b1}≅\frac{4\pi }{3}R_{1,\max}^{3}P_{0},$$

**Fig. 13:**
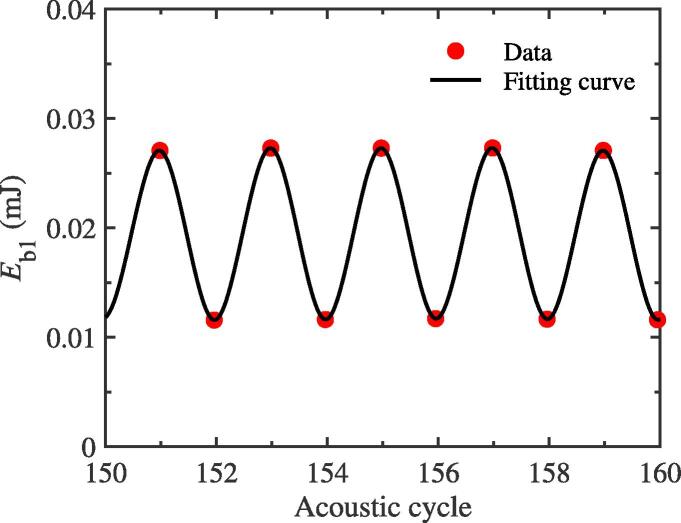
Time-varying energy of cavitation bubble 1 for the two-bubble case. The equilibrium radii of bubbles are both 10 μm. The driving amplitude and frequency, $p_{a}$ and *f*, are 1.35 atm and 43 kHz, respectively. The red solid circles are the results calculated by using Eq. (7), and the black line is the correspondin.g fitting curve.

When the parameters used to simulate remain the same as those in Fig. 2(a), Fig. 13 shows the variation of energy of bubble 1 as time goes on while the f/2 SH component generates. Obviously, the energy value of bubble 1 changes between two different peaks 0.012 and 0.027 mJ. The energy change trend of bubble 2 is similar to that of bubble 1, which is no longer given here. By Fourier spectrum analysis of this energy curve, SH component, f/2, can be obtained, which is consistent with that in Fig. 13 and is no longer presented in this subsection. In essence, the generation of SH in energy curve is also related to the interaction between bubbles. From Eq. (7), we can see that the energy of cavitation bubble is related to the maximum value of pulsation per acoustic cycle. And from the previous subsection, it can be seen that the interaction between bubbles leads to the difference in the peak value of pulsation, which is the cause of the generation of SH. Therefore, in each acoustic period, the alternating change of energy of bubble 1 is easily understood qualitatively.

## 6 Conclusion

In the paper, the 1/2 order SH wave of two interacting cavitation bubbles is investigated numerically by using Fourier spectrum analysis. Firstly, comparing with the single bubble dynamics, we find that the f/2 SH wave in the case of two bubbles could be affected by the interaction between bubbles. This mutual interaction can sometimes promote the production of f/2 SH, and sometimes inhibit the appearance of f/2 SH. Secondly, the driving amplitude and the distance between bubbles have effects on the f/2 SH. The higher the driving amplitude, the larger the mutual interaction is, and also the greater the degree of promotion or suppression of the f/2 SH is. Similarly, as the distance between bubbles decreases, the mutual interaction will increase, and the promotion or suppression of the f/2 SH will also increase. The change of mutual interaction can be known from the radiative pressure between bubbles. It should be pointed out that, while the distance between bubbles is fixed and the driving amplitude exceeds a certain value, the bubble pulsation will appear the 1/4 order SH component, f/4. If the driving amplitude continues to increase, the pulsation will eventually tend to be chaotic, leading to bubble destruction. The f/4 SH and chaos are not the topics discussed in this paper and will be studied in another article. Thirdly, when the f/2 SH occurs, by analyzing the temperature evolution inside the pulsating bubble, we find the instant temperature in the bubble for the two-bubble case would vary between two different peaks. Furthermore, as time goes on, the energy of cavitation bubble would also alternate change between two different peak values.

Compared with Ref. [25], our work focuses on the generation of f/2 SH affected by mutual interaction between bubbles. Although the f/2 SH is used in many fields as an indicator for stable cavitation, as matter of fact, the interaction between the cavitation bubbles that affect the SH is very complex, especially when the number of bubbles is relatively large. We have qualitatively analyzed the production of f/2 SH for the two-bubble case at present, and this interesting phenomenon still needs further investigations.

## Data availability

The data that support the findings of this study are available from the corresponding author upon reasonable request.

## CRediT authorship contribution statement

**Feng Tao:** Conceptualization, Methodology, Software, Data curation, Writing – original draft. **Guo-ying Zhao:** Writing – review & editing. **Wei-zhong Chen:** Methodology, Funding acquisition, Supervision. **Duo Tao:** Writing – review & editing.

## Declaration of competing interest

The authors declare that they have no known competing financial interests or personal relationships that could have appeared to influence the work reported in this paper.
